# DNA methylation profiling of genomic DNA isolated from urine in diabetic chronic kidney disease: A pilot study

**DOI:** 10.1371/journal.pone.0190280

**Published:** 2018-02-20

**Authors:** Ashani Lecamwasam, Alexandra Sexton-Oates, Jake Carmody, Elif I. Ekinci, Karen M. Dwyer, Richard Saffery

**Affiliations:** 1 Clinical and Disease Epigenetics Group, Murdoch Childrens Research Institute, Victoria, Australia; 2 Department of Endocrinology, Austin Health, Victoria, Australia; 3 School of Medicine, Faculty of Health Deakin University, Victoria, Australia; 4 Department of Paediatrics, University of Melbourne, Victoria, Australia; Chung-Ang University, REPUBLIC OF KOREA

## Abstract

**Aim:**

To characterise the genomic DNA (gDNA) yield from urine and quality of derived methylation data generated from the widely used Illuminia Infinium MethylationEPIC (HM850K) platform and compare this with buffy coat samples.

**Background:**

DNA methylation is the most widely studied epigenetic mark and variations in DNA methylation profile have been implicated in diabetes which affects approximately 415 million people worldwide.

**Methods:**

QIAamp Viral RNA Mini Kit and QIAamp DNA micro kit were used to extract DNA from frozen and fresh urine samples as well as increasing volumes of fresh urine. Matched buffy coats to the frozen urine were also obtained and DNA was extracted from the buffy coats using the QIAamp DNA Mini Kit. Genomic DNA of greater concentration than 20μg/ml were used for methylation analysis using the HM850K array.

**Results:**

Irrespective of extraction technique or the use of fresh versus frozen urine samples, limited genomic DNA was obtained using a starting sample volume of 5ml (0–0.86μg/mL). In order to optimize the yield, we increased starting volumes to 50ml fresh urine, which yielded only 0–9.66μg/mL A different kit, QIAamp DNA Micro Kit, was trialled in six fresh urine samples and ten frozen urine samples with inadequate DNA yields from 0–17.7μg/mL and 0–1.6μg/mL respectively. Sufficient genomic DNA was obtained from only 4 of the initial 41 frozen urine samples (10%) for DNA methylation profiling. In comparison, all four buffy coat samples (100%) provided sufficient genomic DNA.

**Conclusion:**

High quality data can be obtained provided a sufficient yield of genomic DNA is isolated. Despite optimizing various extraction methodologies, the modest amount of genomic DNA derived from urine, may limit the generalisability of this approach for the identification of DNA methylation biomarkers of chronic diabetic kidney disease.

## Background

Diabetes Mellitus (DM) both Type I and Type II, and associated micro and macrovascular complications, have attained pandemic proprotions with a global estimate of 415 million people affected[1]. In Australia, approximately 1.7 million Australians have been diagnosed with diabetes[2]. More than 40% of DM patients develop diabetic nephropathy (DN) which globally remains the leading cause of end stage renal disease (ESRD), often requiring long-term dialysis[3]. The risk of cardiovascular disease (CVD) increases for patients with DN and is the leading cause of mortality[4].

At present the causes of DM remain unclear, but are thought to involve both genetic and environmental determinants. At the interface between genes and environment, epigenetic variation is a potential key player in both DM and associated comorbidities[5]. DNA methylation (DNAm) is the most widely studied epigenetic mark and variations in DNAm profile have been implicated in both Type I and Type II DM[6]. Previous studies have assessed DNAm in whole blood and saliva samples from patients with DN [7, 8] revealing differential DNAm in a number of key genes such as *AKR1B1* and *MTHFR* which have been linked with DN.

DNAm variants in specific genes have potential not only to inform research on diabetic nephropathy but could potentially also provide valuable biomarkers for identifying patients with diabetes at risk of progressive kidney dysfunction. At present such patients are identified by proteinuria, however at this stage it is often too late for effective intervention as the endothelial dysfunction and inflammatory milieu is already established and thus the cardiovascular risk conferred.

Urine is collected rapidly, non-invasively, cheaply and easily. Given its ready availability and passage through the diseased kidney, where cells and cellular debris associated with kidney dysfunction are located, urine represents a potentially valuable source of genomic DNA for measuring DNA methylation profile directly related to kidney disease. Indeed, apart from proteins, urine also contains nucleic acids derived from epithelial cells lining the urinary tract, leukocytes and even malignant cells (in the case of cancers) [9]. However, nucleated cells are typically of low abundance relative to other samples such as blood. In comparison to blood, urine contains fewer interfering proteins and PCR inhibitors and is less infectious for many pathogens[10].

## Objectives

In order to investigate the potential for DNAm analysis in diseases such as DN, we performed a pilot project, characterising typical genomic DNA yield from urine and quality of derived methylation data generated from the widely used Illuminia Infinium MethylationEPIC Beadchip platform (HM850K) relative to blood buffy coat samples.

## Methods

### DNA extraction from urine

**Fig 1:**
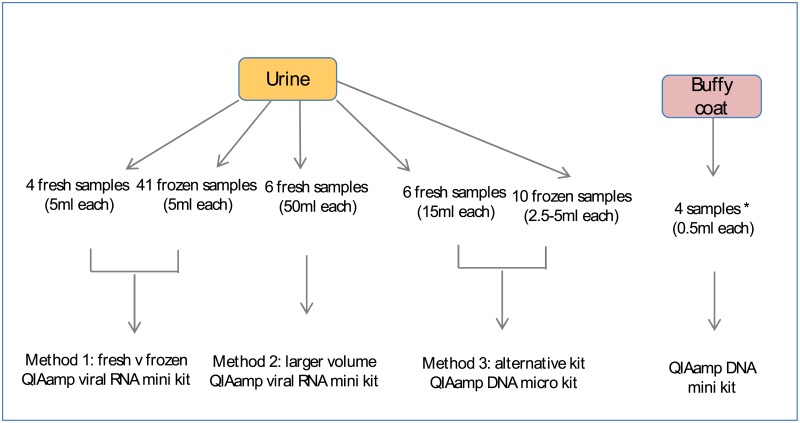
Strategies for optimising gDNA isolation from urine. The impact of several variables on the yield (quantity and quality) of genomic DNA from urine were tested in the current study. These included fresh vs frozen urine, starting volume and isolation kit. For comparison, buffy coat gDNA was isolated using a standard commercially available kit. *Two buffy coat samples matched to two corresponding frozen urine from Method 1.

Three methods were used to extract genomic DNA from urine samples, yields were also compared between fresh and frozen urine (Fig 1).

#### Method 1: Use of frozen versus fresh urine samples

A collection of 41 frozen urine samples, of 5ml aliquots each, were obtained from the Heidelberg Repatriation Hospital biobank, Victoria. Patient cohorts were as follows; Controls (estimated glomerular filtration rate, eGFR > 60ml/min and no albuminuria); Group 1 (eGFR< 60ml/min and no albuminuria); Group 2 (eGFR< 60ml/min and albuminuria) and Group 3 (eGFR >60ml/min and albuminuria). These were processed after long-term storage at -20°C and short term storage at -80°C. Use of biobank samples was approved by the Austin Health Research Ethics Committee (HREC), HREC Reference Number HREC/17/Austin/166. Patients had provided written consent for storage of their samples in the biobank.

Fresh samples (5ml) from three healthy controls (eGFR > 60ml/min and no albuminuria) and one sample from Group 2 (eGFR< 60ml/min and albuminuria), were also analysed. All four samples were first morning urine collections that were kept at room temperature after collection.

DNA was extracted from urine using the commercial QIAamp Viral RNA Mini Kit (QIAGEN), according to manufacturer’s instructions. This has been shown to provide the highest yield and purity of DNA from urine in a comparative analysis of seven tested methods[9]. The urine samples were initially thawed for 1 hour at ambient room temperature. 5ml samples were subsequently transferred into 15ml falcon tubes and centrifuged at room temperature at 500rcf for twenty minutes. The supernatant was discarded and the remaining pellets were resuspended to a final volume of 140μL of Buffer AVL. DNA was eluted in 20μL (frozen urine) or 60μL (fresh urine) of Buffer AVE. DNA concentration was assessed using 1μL by Qubit dsDNA BR Assay kit (Invitrogen by Thermo Fisher Scientific) with repeat measures of 10μL if the original failed to yield measureable DNA amounts.

#### Method 2: Increased starting urine volume

To optimize DNA yield, the starting volume of another six fresh urine samples, from adult volunteers, was increased to 50ml using the same commercial QIAamp Viral RNA Mini Kit, according to manufacturer’s instructions. The samples were transferred into 50ml falcon tubes and centrifuged at 20°C at 600rcf for twenty minutes. The supernatant was discarded and the remaining pellets were resuspended to a final volume of 280μL with a proportionate increase of 1120μL Buffer AVL-carrier RNA as per the protocol. To ensure greater cell lysis, 20μL of proteinase K was added to these six fresh urine samples and incubated at 56°C for one hour. Proportionate volumes of 1120μL of absolute ethanol were added to each of the 280μL samples as per the protocol. DNA was eluted in 60μL of buffer AVE and the DNA concentration was assessed by Qubit dsDNA BR Assay kit, using 10μL of the fresh urine samples.

#### Method 3: Alternative genomic DNA extraction kits

A further 10 frozen urine samples, 2.5-5ml aliquots each, were obtained from the Heidelberg Repatriation Hospital biobank, Victoria. Another 6 fresh, first morning urine samples (15ml) from healthy adult volunteers were obtained.

DNA was extracted from urine using the commercial QIAamp DNA Micro Kit (QIAGEN), according to manufacturer’s instructions. The 10 frozen urine samples were initially thawed for 1 hour at ambient room temperature. All samples were transferred into 15ml falcon tubes and centrifuged at 21°C at 350rcf for five minutes prior to the commencement of the protocol. The supernatant was discarded and the remaining pellets were resuspended to a final volume of 500μL in Buffer AW2. DNA was eluted in 40μL Buffer AE and DNA concentration assessed by Qubit dsDNA BR Assay kit.

#### DNA extraction from buffy coats

Matched buffy coats (BC, n = 4) to the frozen urine used in extraction method 1 were also obtained and DNA was extracted from the buffy coats using the QIAamp DNA Mini Kit (QIAGEN) according to manufacturer’s instructions. DNA was eluted in 200μL of Buffer AE and incubated for 5 minutes at room temperature (15–25°C) prior to centrifugation as per the protocol.

#### DNA methylation profiling

Genomic DNA of greater concentration than 20μg/ml (100-300ng total) from 3 matched urine and buffy coat samples, together with 1 unmatched buffy coat and 1 unmatched urine (total 8 samples), were sent for methylation analysis at the Australian Genomics Research Facility (AGRF) using the Illumina Infinium MethylationEPIC BeadChip Kit (HM850K). This platform interrogates over 850,000 methylation sites quantitatively across the genome at single-nucleotide resolution.

Genome-wide DNA methylation was measured on bisulphite converted genomic DNA using the Illumina Infinium MethylationEPIC BeadChip Array (EPIC) at the Australian Genome Research Facility. Data were processed using the *lumi* and *minfi* packages for R (www.r-project.org/) and normalised using SWAN [11]. Samples with a mean detection p-value ≥ 0.01 were removed (two samples). Probes on the X and Y chromosomes, associated with single nucleotide polymorphisms (minor allele frequency > 1%), or which failed in one or more samples were removed, leaving data for 807,483 probes for subsequent analysis.

## Results

### DNA isolation

Irrespective of extraction technique, starting urine volume or the use of fresh vs frozen urine samples, DNA yield was highly variable between samples. Method 1, the QIAamp viral RNA mini kit, resulted in yields between 0 and 51ng from 5mL of fresh urine, and between 0 and 2,320ng from 5mL of frozen urine. Of these, no fresh urine and only four frozen samples had sufficient yield for DNA methylation analysis (≥500ng).

In order to improve yield, a second method using the QIAamp viral RNA mini kit, this time with a 50mL starting volume, was trialled. DNA from six fresh urine samples was extracted which yielded between 0 and 580ng of DNA. An alternative extraction kit, the QIAamp DNA micro kit was then trialled (Method 3). This resulted in yields between 0 and 708ng from 15mL of fresh urine, and 0 to 64ng from 2.5-5mL of frozen urine.

### DNA methylation (DNAm) profiling

Three matched pairs of urine and buffy coat samples (6 in total), together with one unmatched urine and one unmatched buffy coat sample were profiled using the EPIC array. Following quality control, data from two urine samples were removed leaving a total of six samples, each with data for 807,483 probes for downstream analysis (S1 Table). Principal component analysis, using the *WGCNA* package for R, was undertaken to identify potential known features contributing to variance in the data. This identified individual, sample type, chip position and disease group to be significantly associated with DNA methylation profile.

**Fig 2:**
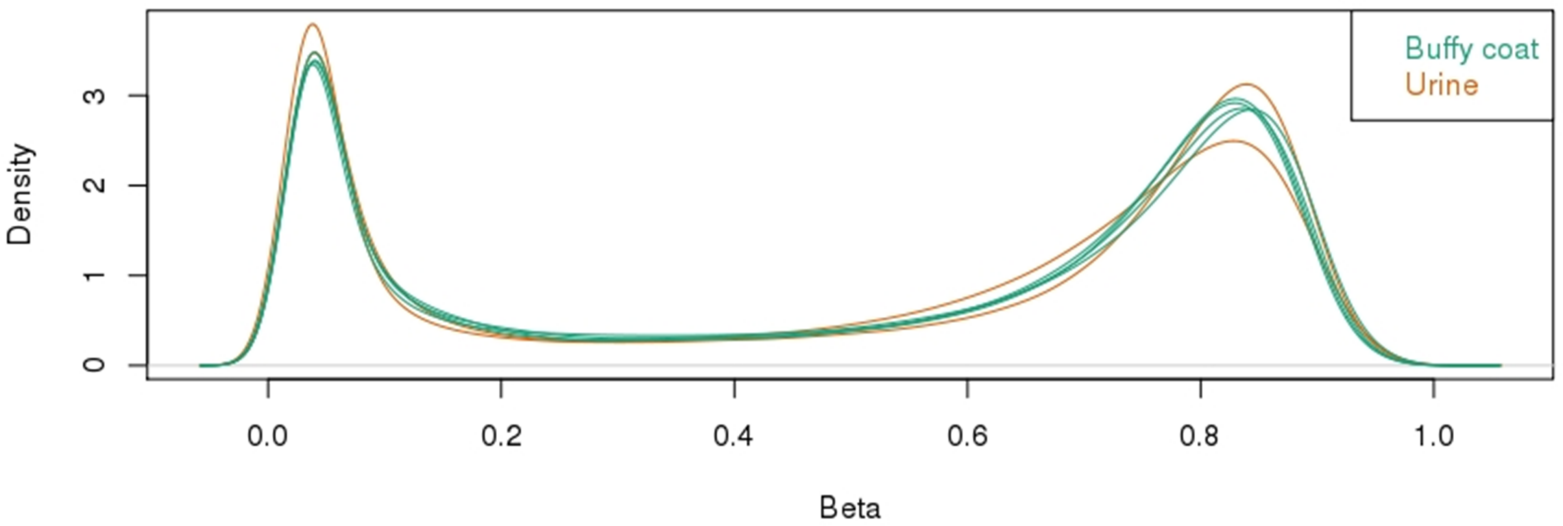
HM850K methylation profiling of buffy coat and urine gDNA. Distribution of all methylation values showing a typical bimodal pattern of methylation associated with the majority of being completely unmethylated or completely methylated.

**Fig 3:**
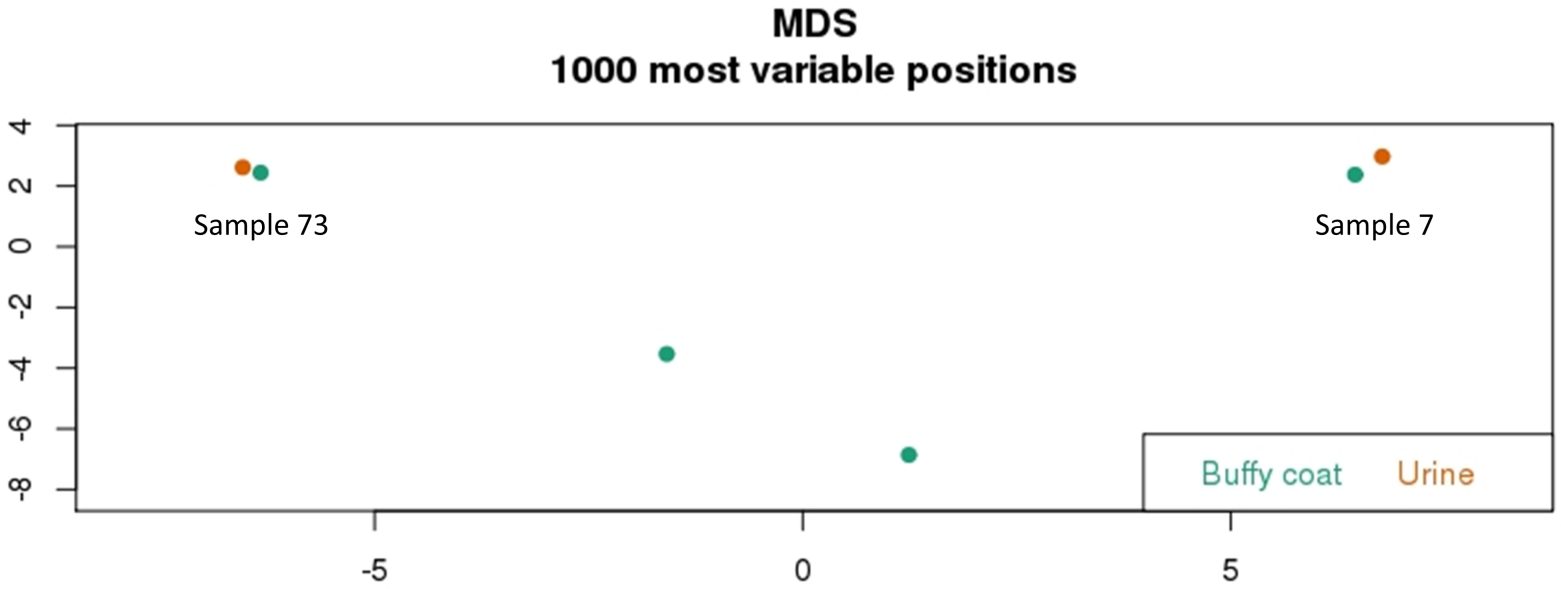
HM850K methylation profiling of buffy coat and urine gDNA. Multidimensional Scaling Plot analysis showing the relationship between samples for the most variable 1000 probes within the dataset.

Preliminary analysis of methylation for each sample revealed a typical bimodal distribution, with the majority of probes either hypomethylated (<0.2 or <20% methylation) or hypermethylated (>0.7 or >70% methylation) (Fig 2). This was very consistent for both buffy coat and urine samples. A ranked list of probes was generated from the most to least variable within the dataset and the top 1000 probes used in multidimensional scaling analysis. It is interesting to observe that despite the variable and low yields of gDNA extracted from urine, the methylation data that we obtained demonstrated that there is minimal difference between the thousand most variable positions in both the matched urine and buffy coat samples of two patients sample 73 and 7 (Fig 3).

**Fig 4:**
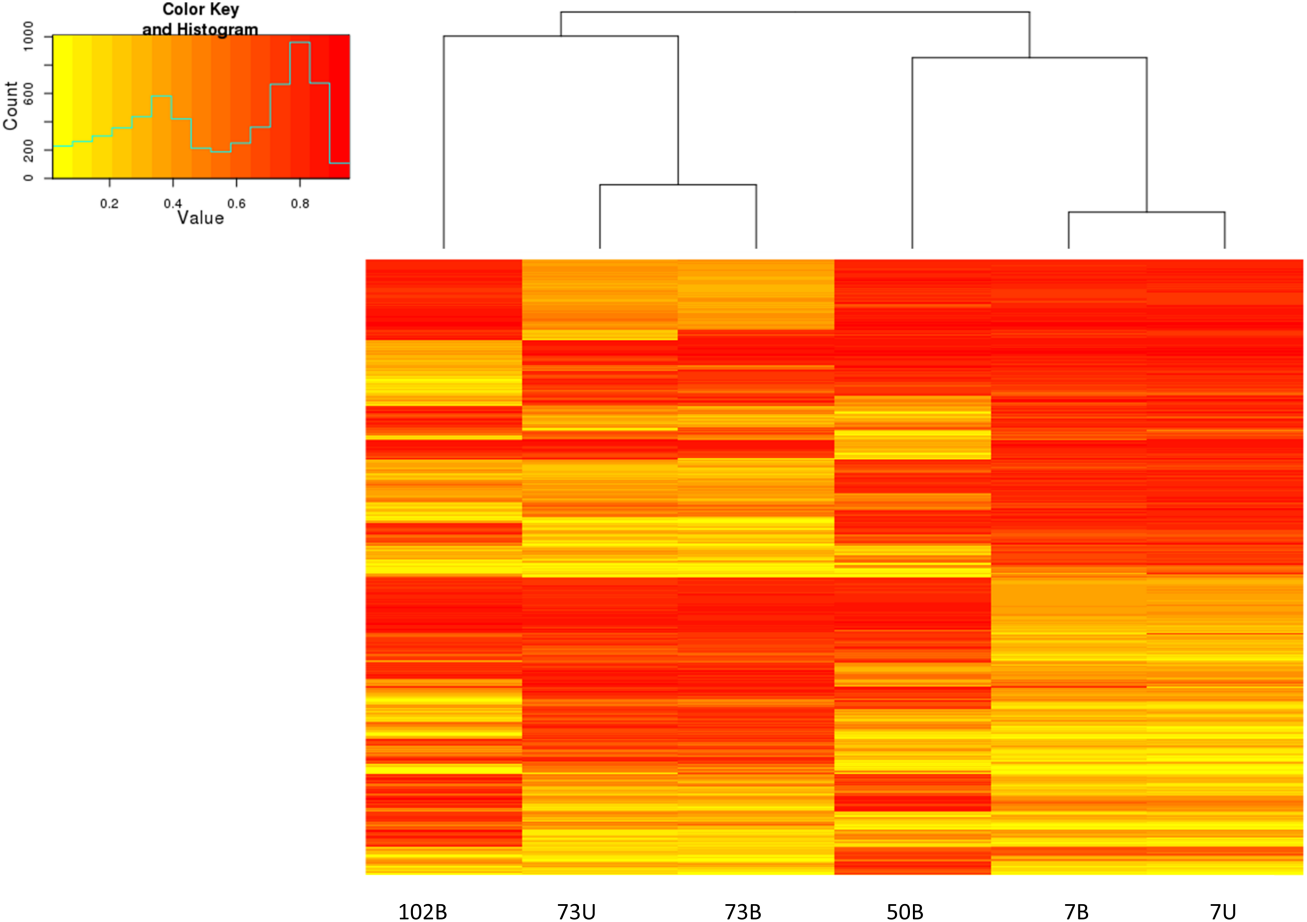
HM850K methylation profiling of buffy coat and urine gDNA. Hierarchical clustering and heatmap of methylation for 1000 most variable probes. Yellow–unmethylated, red–fully methylated.

This is further illustrated, in Fig 4, by the similar methylation profiles observed by hierarchical clustering and heatmaps of the same set of probes for matched samples of patient 73 and 7. Fig 4 also reflects large differences in methylation levels at a subset of probes that distinguish between individuals.

**Fig 5:**
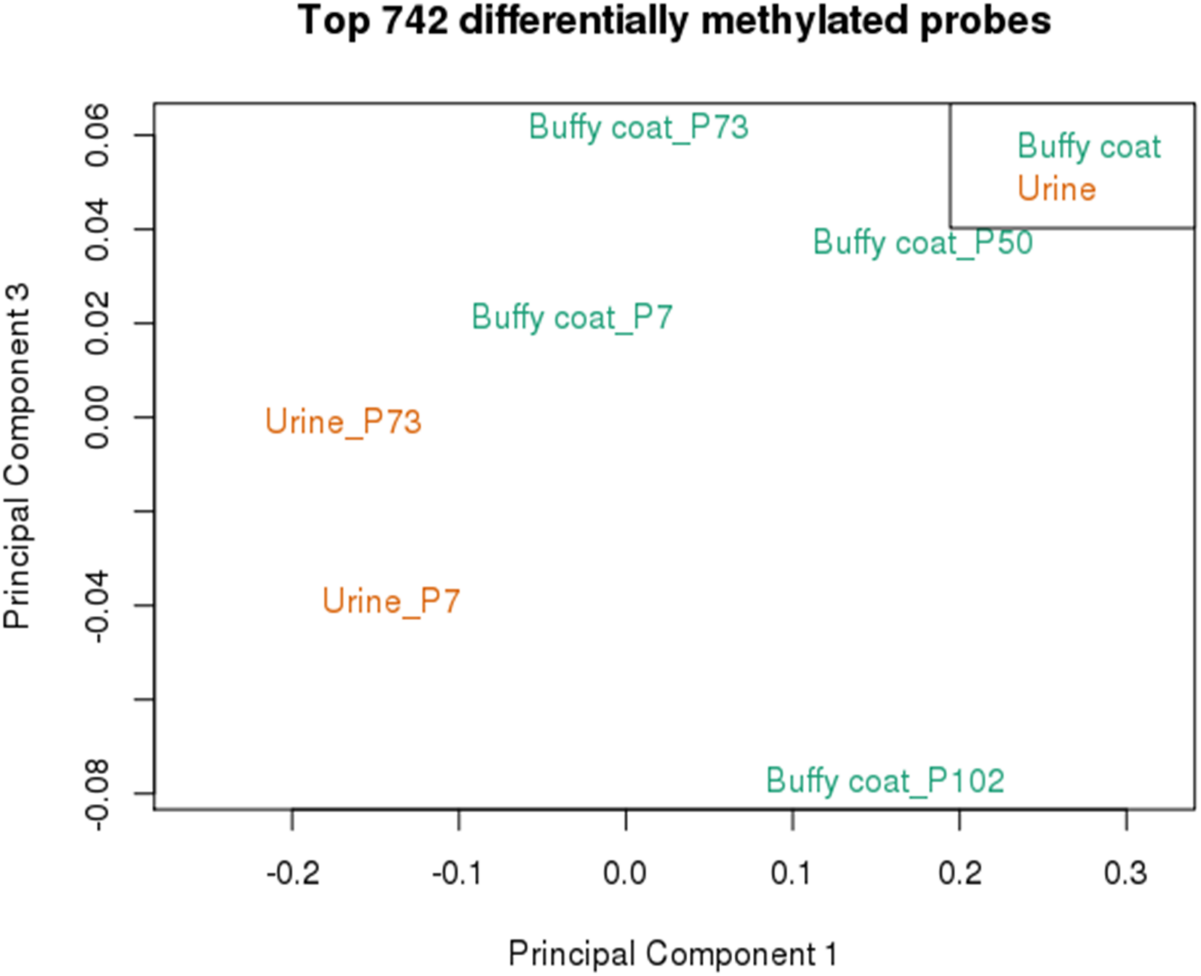
HM850K methylation profiling of buffy coat and urine gDNA. Multidimensional Scaling Plot analysis of top 742 differentially methylated probes, separated by tissue type.

**Fig 6:**
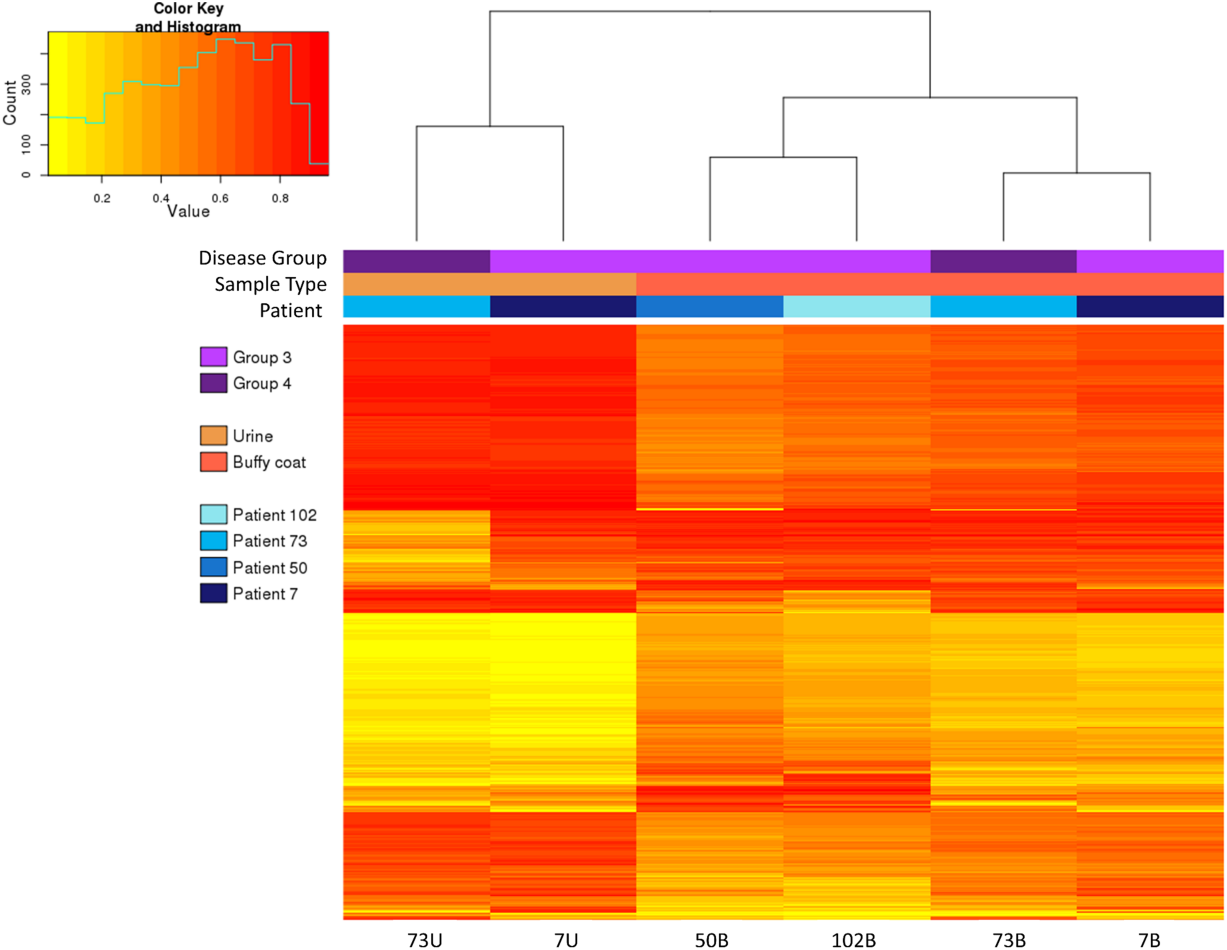
HM850K methylation profiling of buffy coat and urine gDNA. Heat map of methylation for 742 most differential probes by tissue type.

In order to determine the feasibility of identifying urine specific DNA methylation patterns, the top differentially methylated probes between urine and buffy coat were determined by calculating average beta value across all samples of the same type, then calculating delta beta between the two groups. This identified 742 probes with ≥ 20% difference in methylation between urine and buffy coat samples. Data from this subset of probes was sufficient to separate the various datasets according to sample type by multidimensional scaling analysis (Fig 5). This included probes that both showed higher and lower methylation in urine relative to blood (Fig 6).

## Discussion

In order to investigate the potential for DNAm analysis in diabetic chronic kidney disease, we performed this pilot project, attempting to characterise typical genomic DNA yield from urine and the quality of derived methylation data generated from the widely used Illuminia Infinium MethylationEPIC platform.

In our attempts to optimize the yield of DNA extraction from urine we trialled several methods including the use of different commercial kits. Further, we explored the potential for freezing to impact DNA yield by comparing thawed and freshly collected urine samples. Generally speaking, most urine samples gave extremely low yields of genomic DNA irrespective of sampling and storage conditions/time or DNA extraction method. Some of the possible reasons for this may be the inherent low numbers of cells in urine relative to other biospecimens. In the literature, it has been reported that most studies on urine specimens have been performed on the first morning urine, as this is the most cell concentrated[12]. Unfortunately, in practice, first morning urine is not always readily available. However, even the fresh samples that were collected as first morning urine did not yield adequate DNA. The retrospective samples in our cohort were collected as 24hr urine collections. It is possible that when aliquoting initial 10ml samples, the urine was not thoroughly mixed and the sediment containing the cells may have been left behind with only the supernatant being aliquoted. For preservation purposes an additive of 5M sodium hydroxide (NaOH), with a 1:200 dilution (50μl in 10ml) was used to alkalise the urine pH. This is done to preserve albumin, as albumin is known to degrade over long term storage. These urine samples were stored at -20C for at least 5 years. Storing at -20C and not -80C may contribute to cellular degradation in these older samples. Interestingly however, fresh urine samples using the same protocol didn’t provide adequate genomic DNA either.

Preliminary clustering of DNA methylation revealed remarkably similar values for urine and matched blood from two individuals for the 1000 most variable probes (Fig 3). Although this suggests that urine may be a good surrogate for blood in determining a methylation profile in diabetic CKD patients, it is equally likely that this effect is driven by underlying genetic variation at CpG sites, specific to each individual that manifests as large shifts in DNA methylation status following bisulfite conversion and Infinium EPIC analysis. An alternative approach to delineate methylation specific to urine produced less clustered results, likely more indicative of bona fide (albeit of lower magnitude) DNA methylation variation between sample types (Fig 5). It will be interesting in future to further explore the potential for urine-specific DNA methylation markers, particularly in pathological conditions such as CKD and to relate these to clinical parameters.

Although the sample size is far too small to make conclusions based on clinical parameters, it is interesting to note that patients 73 and 7 who demonstrated similar overall methylation patterns in their respective urine and buffy coat samples (Fig 4) nevertheless showed considerable methylation variation at a smaller subset of probes, particularly in urine. Patient 73 was derived from Group 3 (eGFR>60, albuminuria) and patient 7 from Group 2 (eGFR < 60, albuminuria). Furthermore the albuminuria in patient 73 was quantified by an albumin excretion rate (AER) of 58.4mg/24 hours, while patient 7 had an AER of 1133mg/24 hours. It is uncertain whether the more impaired renal function and greater albuminuria in patient 7 compared to patient 73 is contributing to the different methylation patterns between the urine samples of these 2 patients.

Despite analysing close to seventy urine samples, a significant limiting factor in this study was that the majority of the urine samples assayed were sourced for clinical purposes and were therefore not frozen down in a standardised manner. Thus, the findings are potentially confounded by heterogeneity with respect to collection and storage variables. Furthermore, only a small volume of the stored samples were available for analysis. Moving forward, a prospective study may yield more reproducible data.

## Conclusions

Emerging evidence suggests that epigenetic mechanisms, including DNA methylation, may play a key role in the aetiology and progression of diabetes and DN. We have carried out the first study demonstrating the feasibility of genome wide profile of DNAm from urine-derived genomic DNA, collected at different stages of diabetic CKD progression and in normal controls. High quality data can be obtained provided a sufficient yield of gDNA is isolated. However, it is clear that despite optimizing various extraction methodologies as described, as yet unknown factors play a role in modifying the amount of genomic DNA obtainable from urine samples, potentially limiting the generalisability of this approach for the identification of clinically relevant DNAm biomarkers of kidney disease.

## Future Directions

Further studies are required to maximise the yield of genomic DNA obtainable from urine and to identify the factors contributing to the variation in amounts of DNA present in urine. In the interim other biological specimens such as serum or plasma should be investigated to identify DNAm variants associated with diabetic CKD.

## Supporting information

S1 Fig DNA concentration of 5ml fresh urine samples using QIAamp Viral RNA Mini Kit (Elution volume of each sample = 60μL). (PDF)

S2 Fig DNA concentration of 50ml fresh urine samples using QIAamp Viral RNA Mini Kit (Elution volume of each sample = 60μL). (PDF)

S3 Fig DNA concentration of 15ml fresh urine samples using QIAamp DNA Micro Kit (Elution volume of each sample = 40μL). (PDF)

S4 Fig DNA concentration of varying volumes of frozen urine samples using QIAamp DNA Micro Kit (Elution volume of each sample = 40μL). (PDF)

S5 Fig Comparison of total gDNA in urine and buffy coat using method 1. (PNG)

S1 Table gDNA concentration and HM850K probe detection in frozen urine and buffy coat samples. (PDF)

S2 Table Underlying data for generation of S1, S2, S3 and S4 Figs. (XLSX)
