# Supplementary figures and images for: DNA methylation profiling of genomic DNA isolated from urine in diabetic chronic kidney disease: A pilot study
Source: PLoS One. 2018 Feb 20;13(2):e0190280. doi: 10.1371/journal.pone.0190280 (PMC5819761; doi:10.1371/journal.pone.0190280)

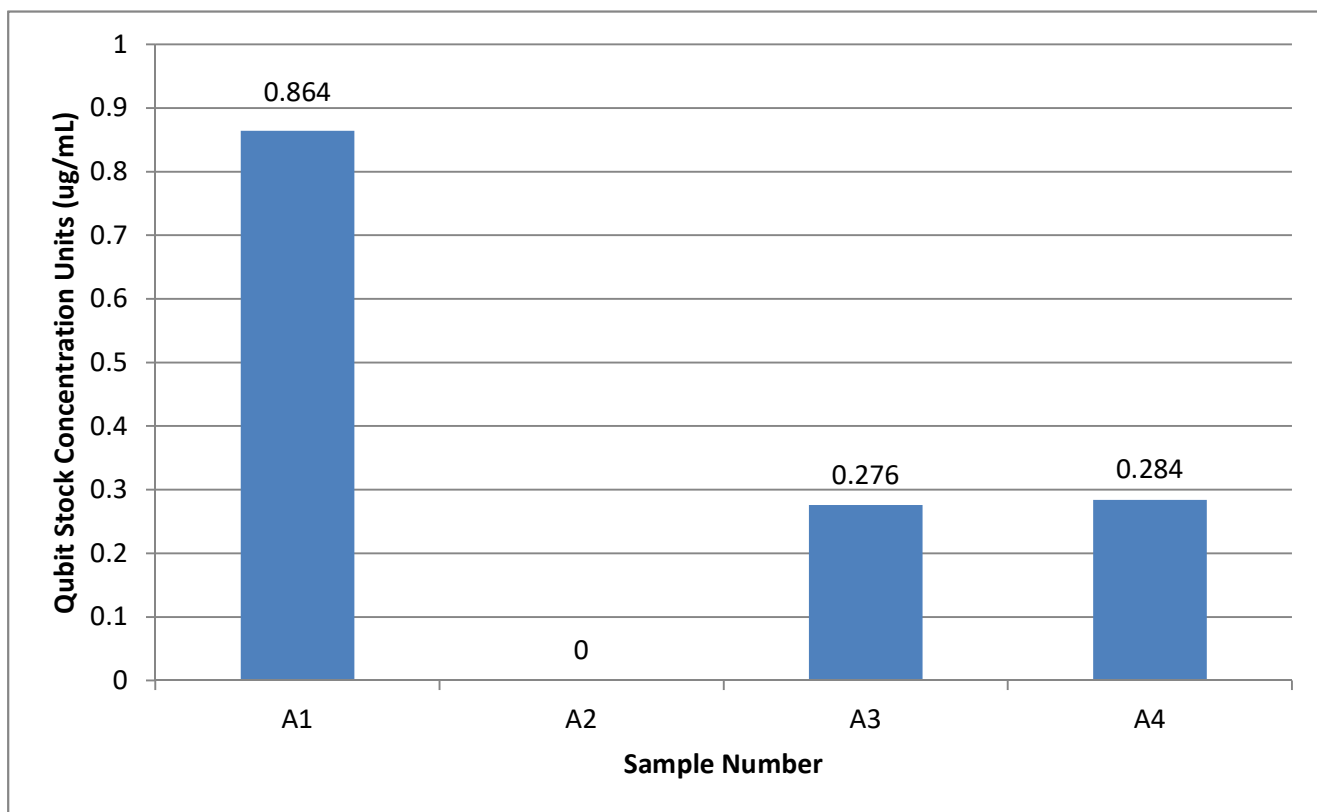

Supplement: S1 Fig — (PDF) [file pone.0190280.s001.pdf]

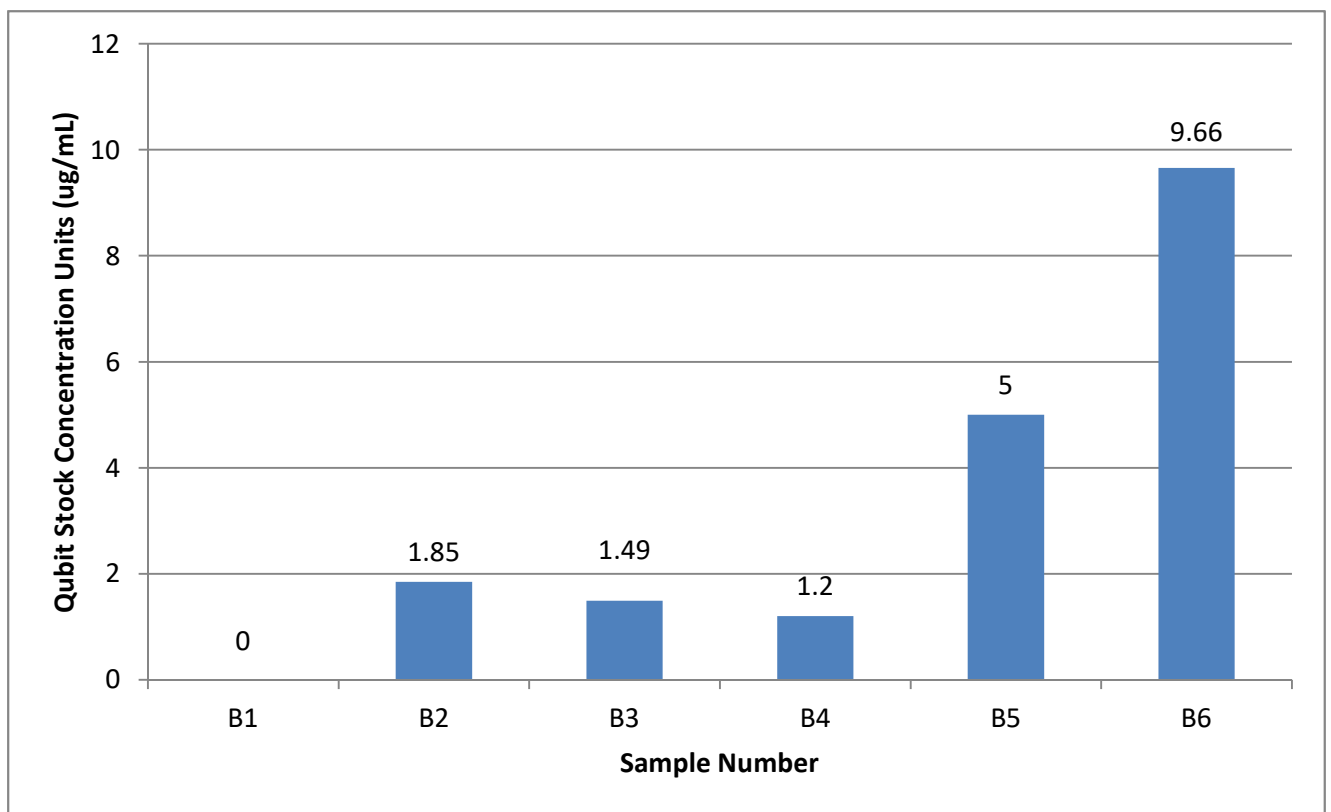

Supplement: S2 Fig — (PDF) [file pone.0190280.s002.pdf]

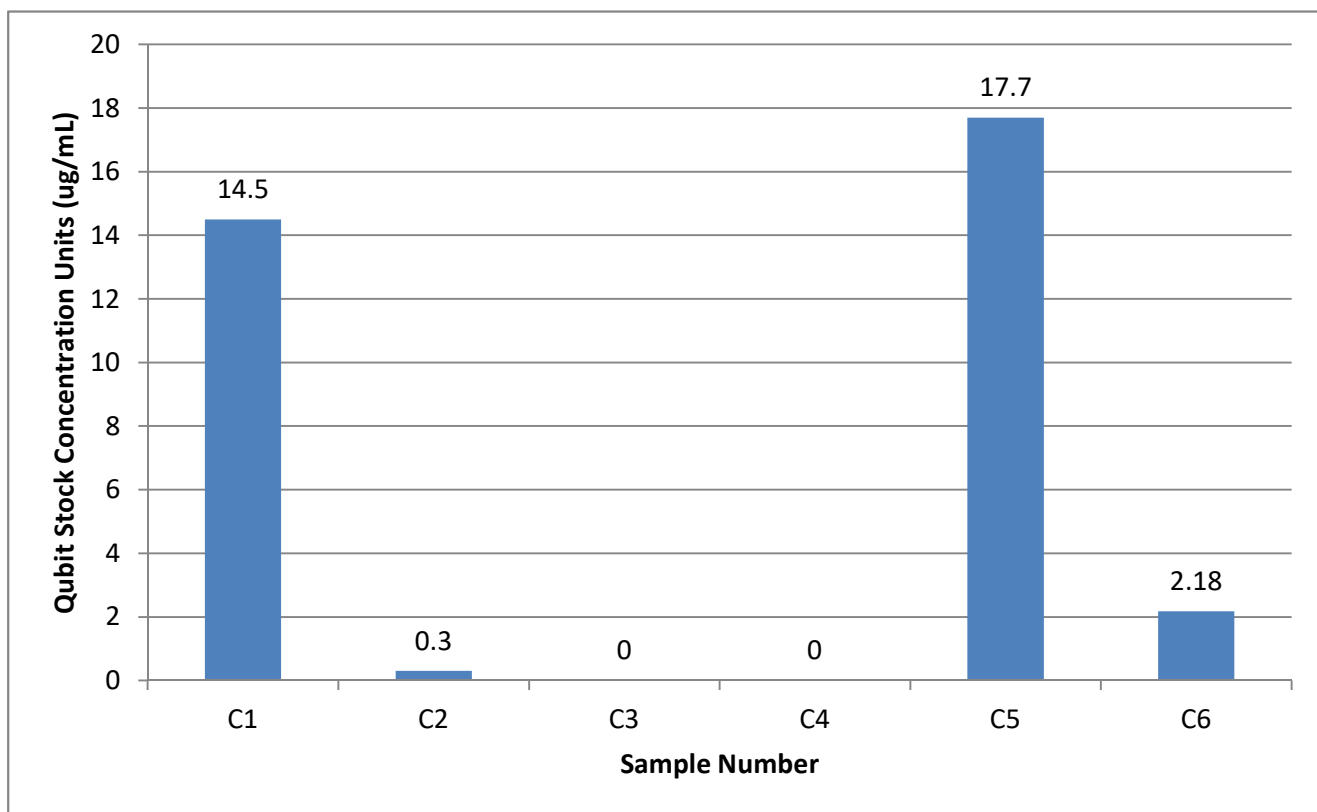

Supplement: S3 Fig — (PDF) [file pone.0190280.s003.pdf]

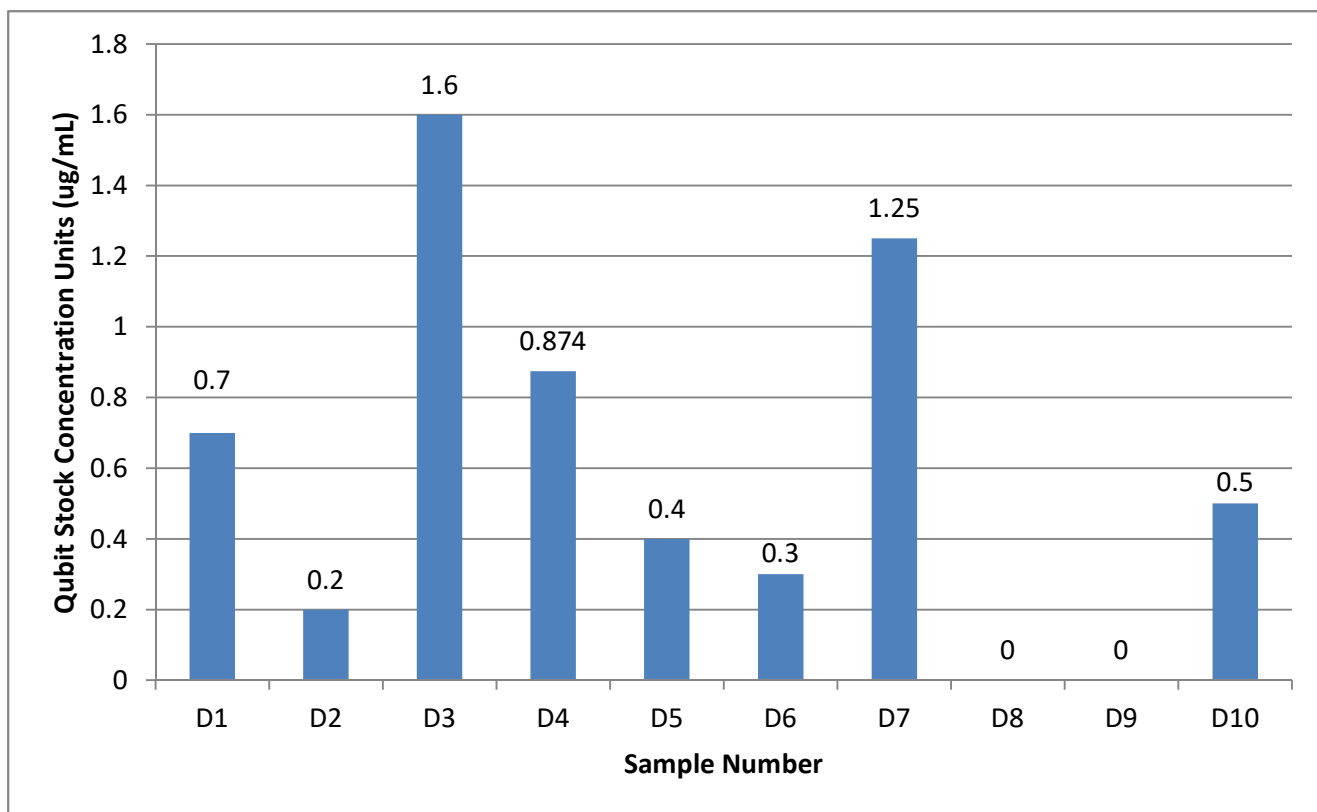

Supplement: S4 Fig — (PDF) [file pone.0190280.s004.pdf]

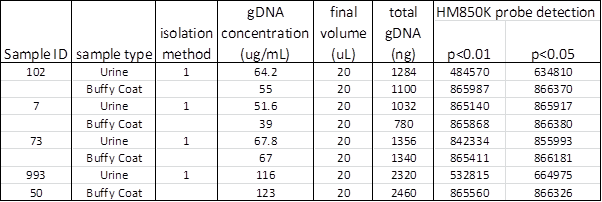

Supplement: S5 Fig — (PNG) [file pone.0190280.s005.png]
