# Supplementary material for: DNA methylation profiling of genomic DNA isolated from urine in diabetic chronic kidney disease: A pilot study
Source: PLoS One. 2018 Feb 20;13(2):e0190280. doi: 10.1371/journal.pone.0190280 (PMC5819761; doi:10.1371/journal.pone.0190280)
Supplement: S1 Table — (PDF) [file pone.0190280.s006.pdf]

Sample ID	sample type	isolation method	gDNA concentration (ug/mL)	final volume (uL)	total gDNA (ng)	HM850K probe detection	
						p<0.01	p<0.05
102	Urine	1	64.2	20	1284	484570	634810
	Buffy Coat		55	20	1100	865987	866370
7	Urine	1	51.6	20	1032	865140	865917
	Buffy Coat		39	20	780	865868	866380
73	Urine	1	67.8	20	1356	842334	855993
	Buffy Coat		67	20	1340	865411	866181
993	Urine	1	116	20	2320	532815	664975
50	Buffy Coat		123	20	2460	865560	866326
